# Prevalence and concentration of Ochratoxin A in beer: A global systematic review, meta‐analysis, and health risk assessment

**DOI:** 10.1002/fsn3.4456

**Published:** 2024-09-10

**Authors:** Yadolah Fakhri, Vahid Ranaei, Zahra Pilevar, Olga F. Belaia, Natalia V. Kolaeva, Mansour Sarafraz, Amin Mousavi Khaneghah

**Affiliations:** ^1^ Food Health Research Center Hormozgan University of Medical Sciences Bandar Abbas Iran; ^2^ School of Health Arak University of Medical Sciences Arak Iran; ^3^ Department of Infectious Diseases of the Institute of Public Health named after F.F. Erisman I.M. Sechenov First Moscow State Medical University of the Ministry of Health of the Russian Federation (Sechenov University) Moscow Russia; ^4^ Environmental and Occupational Health Research Center Shahroud University of Medical Sciences Shahroud Iran; ^5^ Faculty of Biotechnologies (BioTech) ITMO University Saint Petersburg Russia; ^6^ Halal Research Center of IRI, Iran Food and Drug Administration Ministry of Health and Medical Education Tehran Iran

**Keywords:** beer, meta‐analysis, mycotoxin, Ochratoxin A, risk assessment

## Abstract

In the current study, Ochratoxin A (OTA) levels and prevalence in beer were meta‐analyzed, and non‐carcinogenic risk was calculated using the target hazard quotient. Thirty papers with 70 data reports were included in our study. The pooled prevalence of OTA was 50.60%, 95% CI (confidence interval) (36.97–64.19). Five countries with the highest levels of OTA in beer were South Africa (1170.750 μg/L), Slovakia (31.300 μg/L), Portugal (3.140 μg/L), Tunisia (1.052 μg/L), and Greece (0.707 μg/L). The pooled levels of OTA were 0.089 μg/L, 95% CI (0.077–0.101 μg/L). Meta‐regression shows that OTA prevalence in beer decreased over time insignificantly (*p* value: .084). Except in South Africa and Slovakia, consumers in other countries are at an acceptable non‐carcinogenic risk due to OTA in beer. Hence, it is recommended that the quality of raw materials, especially barley, be controlled in the storage and processing conditions in South Africa and Slovakia.

## Graphical abstract

In the current study, levels and prevalence of Ochratoxin A (OTA) in beer were meta‐analyzed and non‐carcinogenic risk was calculated by the target hazard quotient. Thirty papers with 70 data reports were included in our study. The pooled prevalence of OTA was 50.60%, 95% CI (confidence interval) (36.97–64.19). Five countries with the highest levels of OTA in beer were South Africa (1170.750 μg/L), Slovakia (31.300 μg/L), Portugal (3.140 μg/L), Tunisia (1.052 μg/L), and Greece (0.707 μg/L).

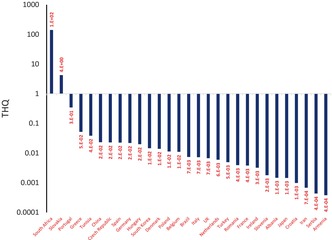

## 1 INTRODUCTION

In recent decades, with the increasing industrialization of developing countries, environmental pollution in water (Talema, 2023), soil (Aralu et al., 2024), air (Feng et al., 2024), and food is increasing (Chen et al., 2023; Gul et al., 2024; Han et al., 2024; Nie et al., 2023; Wang et al., 2024). These contaminants include heavy metals (Nie et al., 2023), pathogens (Pieralisi et al., 2023; Toplu & Tuncer, 2023), and other contaminants (Yang et al., 2024; Zhen et al., 2024). An increase in pollution (Ding et al., 2024; Dou et al., 2023), along with other variables, has caused a rise in non‐communicable diseases (Bahardoust et al., 2023). Mycotoxins are toxic secondary metabolites of certain fungi (Basso et al., 2023; Pires et al., 2022; Thakaew & Chaiklangmuang, 2023; Zamanpour et al., 2023). They can contaminate various agricultural products due to inappropriate harvesting, transportation, storage, and processing practices (Bai et al., 2020, 2022, 2024; Liang et al., 2024; Neme & Mohammed, 2017).

Mycotoxins encompass many compounds, with more than 400 types identified. They are a major concern for food safety and security, as they can result in significant economic losses and pose risks to public health (Khodaei et al., 2021). Ochratoxin A (OTA) is a mycotoxin produced by various fungi, mainly *Aspergillus ochraceus*, *Aspergillus ostianus*, and *Penicillium verrucosum*. It contaminates various food and beverage products, including beer (Mahmudiono et al., 2023; Silva et al., 2020).

Ochratoxin A has been associated with a range of adverse health effects, making it a considerable concern for human consumption. One of the significant health risks related to OTA is its nephrotoxicity, as it has been linked to kidney disease, referred to as Balkan endemic nephropathy (BEN) (Li et al., 2021). Furthermore, it is classified as a potential human carcinogen, particularly associated with kidney cancer, and has raised concerns about its genotoxic effects (Tabarani et al., 2020). The International Agency for Research on Cancer (IARC), which is part of the World Health Organization (WHO), has classified OTA as a Group 2B carcinogen (Ostry et al., 2017). Chronic exposure to OTA has also been implicated in various health conditions, including immune system suppression, developmental abnormalities, and potential impacts on reproductive health (Niaz et al., 2020). The mechanisms of OTA‐induced nephrotoxicity included cell cycle arrest, DNA damage, inhibition of protein synthesis, oxidative stress, and cell apoptosis (Gavahian, 2023;  Khoi et al., 2021). Tolerable intake is the maximum intake of substances in food, such as nutrients or contaminants, that can be consumed, and no significant adverse health effects are observed (EFSA Panel on Contaminants in the Food Chain [CONTAM] et al., 2020). The FAO/WHO Expert Committee on Food Additives (JECFA) established a provisional tolerable weekly intake (PTWI) for OTA in 2001. The PTWI is set at 100 ng/kg‐week (nanograms per kilogram‐week) (Joint FAO/WHO Expert Committee on Food Additives Meeting and World Health Organization, 2001). OTA can contaminate grains and other raw materials used in beer production (Rocha et al., 2023). Beer is one of the oldest and most widely consumed beverages worldwide, with global consumption of about 191 million kiloliters in 2019 (Schabo, Alvarenga, et al., 2021). It is a fermented beverage typically made from malted barley, yeast, hops, and water, although other grains, such as wheat, rice, and corn, can also be used (Dabija et al., 2021). Moreover, beer can be a part of the human diet, but it is important to consume it in moderation and be aware of its potential effects on health (Salanță et al., 2020). The incidence of OTA in beer can vary depending on various factors, such as the quality of the raw materials used, the brewing process, and storage conditions (Schabo, Freire, et al., 2021). The brewing process and fermentation with yeast further reduce the presence of OTA (Benešová et al., 2012). Despite the efforts to minimize OTA contamination during beer production, trace amounts of OTA can pass into beer (Xu et al., 2023). Across the globe, many research studies have explored the occurrence of OTA in beer. One study examined OTA presence in a sample of 106 beers from various European countries. OTA was detected in 68% of the beer samples, with levels ranging from 0.04 to 0.189 μg/L (Bertuzzi et al., 2011). Another study in Tunisia found OTA in 45% of the beer samples tested, with levels ranging from 0.04 to 0.35 μg/L (Lasram et al., 2013). The mean OTA levels in non‐alcoholic beer in local and imported samples in Iran were 0.096 and 0.06 μg/L, respectively (Mahdavi et al., 2007). In Turkey, OTA was detected in 14% of beer samples (levels ranged from 0.012 to 0.045 μg/L) (Kabak & Toxicology, 2009). Another study investigated the occurrence of OTA through Polish beer consumption. OTA was detected in 93% of the beer samples, with mean levels of 0.053 μg/L (Grajewski et al., 2019). Differences in concentration and presence of OTA in beer can be caused by differences in storage conditions of raw materials, the production process, and the quality control of beer in different countries. Several studies have been conducted on the levels and prevalence of OTA in beer (Appendix S1), Therefore, the main aims of the current study were to meta‐analyze the levels of OTA in beer based on country subgroups and to conduct a health risk assessment.

## 2 MATERIALS AND METHODS

### 2.1 Search strategy

This study searched papers on levels and prevalence of OTA in beer based on PRISMA (The Preferred Reporting Items for Systematic Reviews and Meta‐Analysis) guidelines (Selçuk, 2019). Search was conducted in Scopus, Embase, Web of Science, and PubMed until February 15, 2024. Keywords were included: “**Mycotoxins**” OR “**phytotoxin**” OR “**Ochratoxin A**” OR “**OTA**” AND “**alcoholic drinks**” OR “**Beverages**” OR “**beer**” OR “**beer drink**” OR “**Non‐alcoholic drink**” AND “**Occurrence**” OR “**Prevalence**.” The title and abstract of papers were assessed, and related documents were downloaded. Disagreement between authors (VR and AMK) in selecting papers was resolved with the comment of correspondence (YF).

### 2.2 Criteria of study

The criteria in our study were (a) a cross‐sectional study, (b) present mean and standard deviation (SD) or range of levels of OTA, (c) available English full‐text, and (d) valid detection method of OTA. Exclusion criteria were (a) review study, (b) letter to editor, (c) book, (d) thesis, and (e) experimental studies. Country, positive samples, total samples, mean, standard deviation, method of analysis, limit of detection (LOD), and limit of quantification (LOQ) were extracted.

### 2.3 Meta‐analysis

The levels of OTA in beer were meta‐analyzed by mean and standard error, and the prevalence of OTA was meta‐analyzed by metaprop command based on country subgroup (Li et al., 2023). The degree of heterogeneity was calculated using the *I* ^2^ index; when *I* ^2^ >50%, the degree of heterogeneity is high; hence, the random effects model was applied to estimate pooled prevalence and levels of OTA in beer (Bahardoust et al., 2024; Gao et al., 2023). Stata software version 17.0 (College Station, TX, USA) was used for meta‐analysis.

### 2.4 Health risk assessment

The daily intake of consumers due to OTA in beer was calculated as per (Cai et al., 2023; Luo et al., 2022; Xiong, Wen, et al., 2022):

(1) $$\mathrm{EDI}=\frac{C\times \mathrm{IR}\;}{\mathrm{BW}\;}$$

where EDI is the estimated daily intake (μg/kg‐day); *C*, levels of OTA (μg/kg); IR is the ingestion rate of beer (Appendix S2); and BW, body weight for adults is 70 kg (EPA, 2015; Zhang et al., 2024).

The target hazard quotient (THQ) was calculated as per (Bai et al., 2023; Gao et al., 2022; Xiong, Chen, et al., 2022):

(2) $$\mathrm{THQ}=\frac{\mathrm{EDI}\;}{\mathrm{TDI}\;}$$

In this equation, EDI is the estimated daily intake (μg/kg‐day) and TDI is the tolerable daily intake (μg/kg‐day) (Zhu et al., 2024). The TDI for OTA is equal to 0.017 μg/kg‐day (EFSA, 2020), and when THQ is higher than 1 value, the non‐carcinogenic risk is unacceptable (EPA, 2015).

## 3 RESULTS AND DISCUSSION

**FIGURE 1:**
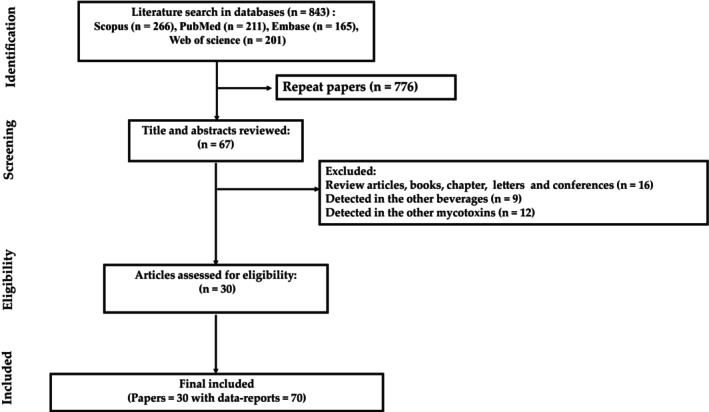
Process of selection papers based on PRISMA.

Our study included 30 papers and 70 data reports (Figure 1; Appendix S1). The countries with the highest prevalence (100%) of OTA in beer were Armenia, the Netherlands, the UK, Kosovo, Macedonia, Montenegro, Russia, Sweden, Ireland, Denmark, Hungary, and Iran (Table 1). The pooled prevalence of OTA was 50.60%, 95% CI (36.97–64.19) (Table 1).

**TABLE 1:** The meta‐analysis prevalence of OTA in beers based on countries (μg/L).

Subgroups	NS^a^	ES^b^, 95% CI	Weight (%)	Heterogeneity statistic	Degrees of freedom	*p* value	*I* ^2^
Italy	5	43.03, 95% CI (29.55–57.02)	7.84	13‐93	4	‐01	71.29%
Morocco	1	0.00, 95% CI (0.00–52.18)	1.35	‐	0	‐	.%
South Africa	3	14.68, 95% CI (0.25–41.51)	4.74	‐	2	‐	.%
Belgium	3	94.18, 95% CI (83.74–99.88)	4.62	‐	2	‐	.%
Brazil	2	6.74, 95% CI (3.06–11.52)	3.18	‐	1	‐	.%
Turkey	3	27.12, 95% CI (6.24–53.51)	4.31	‐	2	‐	.%
Spain	7	71.53, 95% CI (35.88–97.37)	10.53	170‐07	6	0	96.47%
Taiwan	1	0.00, 95% CI (0.00–18.53)	1.53	‐	0	‐	.%
South Korea	1	4.35, 95% CI (0.53–14.84)	1.59	‐	0	‐	.%
Hungary	1	100.00, 95% CI (86.28–100.00)	1.56	‐	0	‐	.%
Japan	2	65.08, 95% CI (49.24–79.50)	3.08	‐	1	‐	.%
Iran	1	100.00, 95% CI (94.87–100.00)	1.6	‐	0	‐	.%
Czech	6	75.72, 95% CI (42.49–98.42)	8.96	128‐23	5	0	96.10%
Albania	1	75.00, 95% CI (19.41–99.37)	1.3	‐	0	‐	.%
Armenia	1	100.00, 95% CI (15.81–100.00)	1.11	‐	0	‐	.%
Bosnia	1	0.00, 95% CI (0.00–97.50)	0.92	‐	0	‐	.%
Croatia	1	18.18, 95% CI (2.28–51.78)	1.48	‐	0	‐	.%
Denmark	1	100.00, 95% CI (47.82–100.00)	1.35	‐	0	‐	.%
France	2	20.27, 95% CI (9.52–33.15)	2.94	‐	1	‐	.%
Germany	2	6.93, 95% CI (2.80–12.26)	3.07	‐	1	‐	.%
Ireland	1	100.00, 95% CI (39.76–100.00)	1.3	‐	0	‐	.%
Kosovo	1	100.00, 95% CI (2.50–100.00)	0.92	‐	0	‐	.%
Macedonia	1	100.00, 95% CI (2.50–100.00)	0.92	‐	0	‐	.%
Montenegro	1	100.00, 95% CI (2.50–100.00)	0.92	‐	0	‐	.%
Poland	3	80.43, 95% CI (47.25–99.91)	4.39	‐	2	‐	.%
Romania	1	54.55, 95% CI (23.38–83.25)	1.48	‐	0	‐	.%
Russia	1	100.00, 95% CI (2.50–100.00)	0.92	‐	0	‐	.%
Serbia	1	25.00, 95% CI (3.19–65.09)	1.43	‐	0	‐	.%
Slovenia	1	50.00, 95% CI (1.26–98.74)	1.11	‐	0	‐	.%
Sweden	1	100.00, 95% CI (2.50–100.00)	0.92	‐	0	‐	.%
Switzerland	1	0.00, 95% CI (0.00–97.50)	0.92	‐	0	‐	.%
Netherlands	1	100.00, 95% CI (15.81–100.00)	1.11	‐	0	‐	.%
UK	1	100.00, 95% CI (15.81–100.00)	1.11	‐	0	‐	.%
China	2	0.00, 95% CI (0.00–0.42)	3.16	‐	1	‐	.%
Cameroon	1	0.00, 95% CI (0.00–23.16)	1.51	‐	0	‐	.%
Tunisia	2	21.58, 95% CI (12.42–32.30)	3.15	‐	1	‐	.%
Malawi	1	0.00, 95% CI (0.00–33.63)	1.45	‐	0	‐	.%
Slovakia	1	87.50, 95% CI (47.35–99.68)	1.43	‐	0	‐	.%
Latvia	1	0.00, 95% CI (0.00–3.62)	1.61	‐	0	‐	.%
Greece	1	35.48, 95% CI (19.23–54.63)	1.57	‐	0	‐	.%
Portugal	1	10.59, 95% CI (4.96–19.15)	1.61	‐	0	‐	.%
Overall	70	50.60, 95% CI (36.97–64.19)	100	2178‐44	69	0	96.83%

^a^ Number study.

^b^ Effect size: pooled concentration.

The finding of a 100% prevalence of Ochratoxin A (OTA) in beer from several countries and a pooled prevalence of 50.60% across different countries suggests a widespread occurrence of this contaminant in beer. Several reasons could contribute to this finding, including (a) agricultural practices, (b) climate and environmental conditions, (c) storage and processing conditions, (d) variations in regulatory standards and detection methods, (e) global trade and raw materials sourcing, and (f) lack of awareness or monitoring (Fakhri et al., 2021, 2022; Mollayusefian et al., 2021). The research by Bellver Soto et al. highlights the impact of environmental conditions on the proliferation of fungi that produce OTA in beer. It points out that the humid and warm climates prevalent in Southern European countries create ideal conditions for the growth of these fungi. Consequently, the climate in Southern Europe, known for its warmth and humidity, provides a suitable habitat for these fungi, promoting their growth and activity. This increases the likelihood of OTA presence in agricultural products from these areas (Bellver Soto et al., 2014). The study conducted by Bertuzzi examined the occurrence of certain mycotoxins in beer samples from various European countries. The survey found no traces of Aflatoxins in the samples, but OTA, Deoxynivalenol (DON), and Fumonisins were detected in many samples at low levels. Notably, there were variations in the presence of these mycotoxins across different European regions. The study noted that the minimal contamination of OTA in Southern European beer might be due to the predominance of *P. verrucosum*, a fungus more common in Northern Europe's cooler, damp environments (Bertuzzi et al., 2011). In these Northern regions of Europe, effective drying methods are crucial to prevent postharvest OTA contamination, as discussed by Magan and Aldred in their research (Magan & Aldred, 2005).

The main source of OTA is contamination by certain species of fungi (like *Aspergillus* and*Penicillium*) that affect crops like barley and wheat, which are commonly used in beer production. Agricultural practices, climate, and storage conditions can influence the prevalence of these fungi. Contamination with OTA is prevalent in many commodities, with particularly high levels found in corn, rye, and coffee (Joint & Additives, 2002). In the Sugita‐Konishi et al. study, OTA was detected in oatmeal, wheat flour, rye, buckwheat flour, beer, and wine but not in rice, corn grits, popcorn grain, canned, or frozen corn. Rye and raisin samples exclusively showed OTA in imported products, while beer, buckwheat flour, and wine contamination was observed in both imported and domestic items. The study emphasizes the significance of conducting a comprehensive survey on OTA contamination in domestic and imported samples (Sugita‐Konishi et al., 2006). It is worth noting that different countries have varying regulations regarding acceptable levels of OTA in food and beverages. The detection and reporting methods can also vary, leading to differences in reported prevalence rates (Duarte, Lino, & Pena, 2010). In some studies, the LOD and LOQ are lower than those of other studies based on their detection method. Therefore, the prevalence in the current studies is expected to be higher than in other studies (Rodríguez‐Cañás et al., 2023). Moreover, in some regions, there might be less awareness or fewer resources dedicated to monitoring and controlling OTA levels in agricultural products and food items like beer (Anli & Alkis, 2010; Ayelign & de Saeger, 2020).

Differences in the methodology employed for analyzing OTA in beer can indeed lead to variations in results. Variances in sample preparation, extraction techniques, detection methods, and quality control measures may influence the reported levels of OTA contamination in beer samples across different studies. For example, ELISA (enzyme‐linked immunosorbent assay) is generally considered more sensitive than TLC (thin‐layer chromatography) for quantitatively measuring OTA in beer. ELISA is a highly specific and sensitive immunological method that can detect lower levels of toxins, providing more accurate quantitative results than TLC. This higher sensitivity makes ELISA a preferred choice in many analytical laboratories for precise measurements of contaminants like OTA in complex matrices such as beer (Mahdavi et al., 2007). ELISA method has a lower detection limit and more sensitivity than TLC (Wolf‐Hall & Bullerman, 1996).

The five countries with the highest levels of OTA in beer were South Africa (1170.750 μg/L), Slovakia (31.300 μg/L), Portugal (3.140 μg/L), Tunisia (1.052 μg/L), and Greece (0.707 μg/L) (Table 2). The pooled levels of OTA were 0.089 μg/L, 95% CI (0.077–0.101 μg/L) (Table 1). The global consumption of beer across continents, such as Europe, America, and Asia, necessitates vigilance about its OTA contamination. This concern is heightened by the fact that in the European Union (EU), beer, cereals, and related products significantly contribute to the daily intake of OTA (Deetae et al., 2013). Chronic exposure to OTA has been implicated in various health conditions, including immune system suppression, developmental abnormalities, and potential impacts on reproductive health (Niaz et al., 2020).

**TABLE 2:** The meta‐analysis concentration of OTA in beers based on countries.

Subgroups	NS^a^	ES^b^, 95% CI	Weight (%)	Heterogeneity statistic	Degrees of freedom	*p* value	*I* ^2^
Italy	5	0.090, 95% CI (0.062–0.118)	12.02	1112.42	4	0	99.60%
South Africa	1	1170.750, 95% CI (977.066–1364.434)	0	0	0	‐	.%
Belgium	3	0.072, 95% CI (0.007–0.137)	6.87	119.81	2	0	98.30%
Brazil	2	0.055, 95% CI (0.051–0.059)	2.48	0.41	1	‐522	0.00%
Turkey	3	0.187, 95% CI (0.119–0.255)	5.04	631.91	2	0	99.70%
Spain	7	0.196, 95% CI (0.131–0.262)	9.67	286.34	6	0	97.90%
South Korea	1	0.200, 95% CI (0.192–0.208)	2.43	0	0	‐	.%
Hungary	1	0.127, 95% CI (0.105–0.149)	2.29	0	0	‐	.%
Japan	2	0.019, 95% CI (0.017–0.022)	4.9	0.13	1	‐724	0.00%
Iran	1	0.260, 95% CI (0.229–0.291)	2.12	0	0	‐	.%
Czech Republic	6	0.071, 95% CI (0.015–0.126)	10.56	308.14	5	0	98.40%
Albania	1	0.015, 95% CI (0.001–0.029)	2.39	0	0	‐	.%
Armenia	1	0.033, 95% CI (0.025–0.041)	2.43	0	0	‐	.%
Croatia	1	0.005, 95% CI (0.001–0.009)	2.45	0	0	‐	.%
Denmark	1	0.101, 95% CI (0.042–0.160)	1.57	0	0	‐	.%
France	2	0.050, 95% CI (0.000–0.132)	4.85	153.09	1	0	99.30%
Germany	2	0.096, 95% CI (0.000–0.252)	4.86	743.56	1	0	99.90%
Ireland	1	0.015, 95% CI (0.007–0.023)	2.43	0	0	‐	.%
Poland	3	0.053, 95% CI (0.013–0.093)	4.17	3.85	2	‐146	48.10%
Romania	1	0.017, 95% CI (0.005–0.029)	2.4	0	0	‐	.%
Serbia	1	0.003, 95% CI (0.001–0.005)	2.46	0	0	‐	.%
Slovenia	1	0.010, 95% CI (0.000–0.026)	2.36	0	0	‐	.%
Netherlands	1	0.042, 95% CI (0.030–0.054)	2.4	0	0	‐	.%
UK	1	0.043, 95% CI (0.000–0.096)	1.69	0	0	‐	.%
China	1	0.354, 95% CI (0.325–0.383)	2.16	0	0	‐	.%
Tunisia	2	1.052, 95% CI (0.000–2.894)	2.31	110.87	1	0	99.10%
Slovakia	1	31.300, 95% CI (18.029–44.571)	0	0	0	‐	.%
Greece	1	0.707, 95% CI (0.578–0.836)	0.66	0	0	‐	.%
Portugal	1	3.140, 95% CI (2.270–4.010)	0.02	0	0	‐	.%
Overall	55	0.089, 95% CI (0.077–0.101)	100	8128.41	54	0	99.30%

^a^ Number study.

^b^ Effect size: pooled concentration.

Various studies, particularly in Europe, have reported OTA contamination in beer. Typically, the levels of OTA in beer are low, ranging from under 0.002 to 2 μg/L (Bertuzzi et al., 2011). However, certain traditional beers from South Africa exhibit notably high OTA levels, with levels reported between 3 and 2340 μg/L (Odhav & Naicker, 2002).

Ezekiel et al.'s research in Africa underscores the risks associated with the improper storage of grains, particularly the increased fungal growth and production of OTA. This issue is compounded during the beer brewing process, where OTA might not be fully eradicated, especially in cases of heavy contamination of raw materials. The study further notes a significant gap in current research on mycotoxins in traditional African beverages, which tends to concentrate on the end products while overlooking the complete process chain from raw materials to finished goods. There is an evident deficiency in understanding the behavior of various mycotoxins throughout the production process and the potential risks they pose to consumers. Despite the prevalent of mycotoxins in raw materials and the high consumption rate of these beverages in numerous households, there are still no targeted regulations to address these concerns (Ezekiel et al., 2018).

Polo's research revealed that barley cultivated in South Africa frequently contains multiple mycotoxins, mainly deoxynivalenol and zearalenone (ZEN), similar to barley from other parts of the world. These toxins were also present in malted barley. Notably, Aflatoxin B1 and Ochratoxin A were detected in all alcoholic beverages made from barley malt, albeit at very low levels. Although the concentration of individual mycotoxins in beer may be low, their cumulative risk may be disastrous and endanger the consumer's health. These findings raise concerns for consumers and suggest that regular mycotoxin analysis of cereal samples is necessary to prevent contamination in the final products of South Africa (Maenetje & Dutton, 2007).

Ochratoxin A (OTA) could be a risk factor for Balkan endemic nephropathy (BEN). Nonspecific symptoms of BEN include fatigue, lethargy, weakness, lumbar pain, pallor, and plantar surfaces. In addition, glomerular and vascular lesions from periglomerular fibrosis and deterioration of the kidneys were observed (Pavlović, 2013; Stiborová et al., 2016). OTA is recognized as the causal factor behind endemic nephropathy and urinary tract tumors in the Balkans (Joint & Additives, 2002). The toxicity of OTA is multifaceted and involves several mechanisms, but common mechanisms include cell death by apoptosis, lipid peroxidation, oxidative stress, and mitochondrial dysfunction (Gupta et al., 2022).

The OTA levels in beer hinge on several crucial factors, with potential contamination during cereal harvest and the established presence of *P. verrucosum* in soil being key contributors. Impurities like dust, weed seeds, and grain fractures can contaminate OTA (Běláková et al., 2011). Elevated moisture levels are a crucial factor in mycotoxin production. Seed and weed admixtures can contribute to higher moisture levels, potentially leading to increased mycotoxin production. Timely clearance and moisture reduction can interrupt the conditions that favor mycotoxin production, contributing to the overall safety of cereals (Tangni & Pussemier, 2006). As stated by Běláková et al., dust can be a direct source of contamination for cereals. It may carry mycotoxins or other contaminants that pose a risk to the quality and safety of cereals. Dust not only directly contaminates cereals but it can also serve as an inoculum. It can introduce microorganisms, such as fungi, which may contribute to mycotoxin production. Moreover, seed and weed admixtures in cereals can have implications for mycotoxin production. These admixtures may temporarily elevate moisture levels, creating conditions conducive to the growth of fungi and subsequent mycotoxin production (Běláková et al., 2011).

During barley cultivation, exposure to environmental conditions conducive to mold growth, such as high humidity, can contaminate OTA. Harvesting practices, including storage conditions postharvest, play a pivotal role in preventing mycotoxin development. Adequate drying and storage practices help minimize the risk of mold growth and mycotoxin production. To prevent mold growth and OTA formation, ensuring proper cereal storage, sufficient aeration, and barley moisture below 13%–14% is essential. Harvesting conditions and air moisture also play a role in influencing OTA formation. According to Gumus et al., heightened OTA levels in barley and malt samples may indicate insufficient storage or processing conditions (Gumus et al., 2004).

In the malting process, where barley undergoes germination and drying, careful attention to hygiene and control of environmental factors is essential. Malt quality assessments should include measures to detect and limit mycotoxin contamination. Implementing effective quality control measures, such as regular testing for mycotoxins like OTA, ensures that only high‐quality malt is used in beer production (Odhav & Naicker, 2002).

By addressing potential sources of contamination at each stage, from barley cultivation to malting, breweries can uphold stringent standards to safeguard beer quality and consumer health. Regular monitoring and adherence to best practices contribute to minimizing the risk of OTA presence in the final beer product (Zinedine & Mañes, 2009). Baxter and colleagues discovered that when Ochratoxin A (OTA) is present in brewing grains, a fraction (13%–32%) of it can also persist in the final beer. However, a considerable amount of OTA is removed during the mashing process. This decrease is mainly due to OTA being converted into the non‐toxic Ochratoxin A by proteolytic enzymes in the mash. Importantly, a significant amount of OTA that is not hydrolyzed remains in the spent grains. OTA also withstands the boiling of the wort, with only slight reductions occurring during fermentation. The findings from these pilot‐scale trials align with those observed in commercial malts and beer (Baxter et al., 2001).

### 3.1 Meta‐regression

**FIGURE 2:**
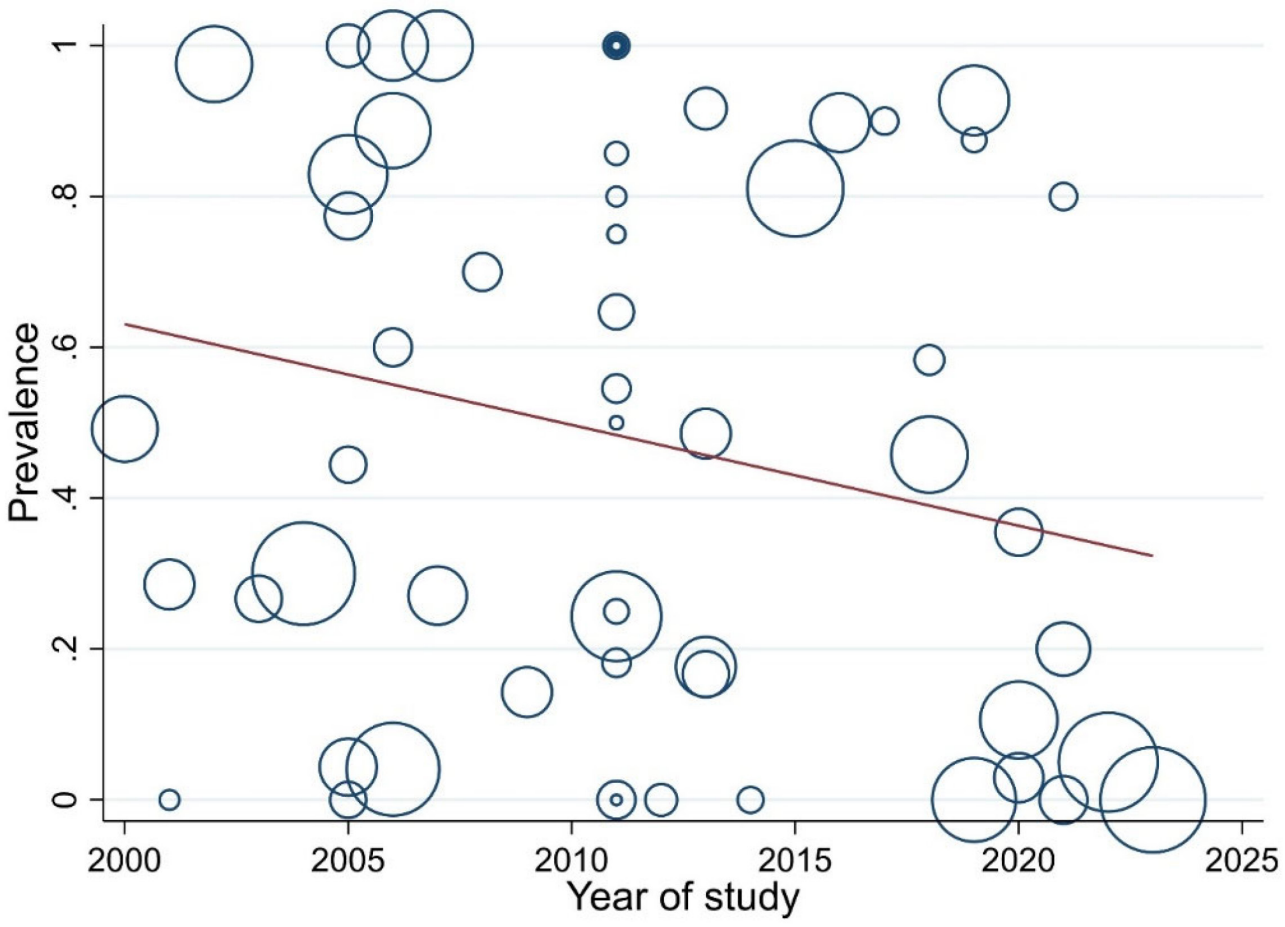
Meta‐regression between prevalence of OTA in beers with over time.

Meta‐regression shows that OTA prevalence in beer decreased insignificantly over time (*p* value = .084) (Figure 2). Brewing practices and quality control measures vary significantly across different breweries and over time. These variations could influence OTA levels in beer. Improvements in brewing technology and quality control might contribute to a decrease in OTA levels, but this effect could be uneven across different studies, contributing to the non‐significant result. Over time, public health agencies worldwide have implemented stricter regulations and standards for food and beverage safety (Ghajarbeygi et al., 2022; Naimi et al., 2022). This includes setting maximum allowable limits for contaminants like OTA in consumable products. Enhanced public health policies ensure producers adhere to safer, more hygienic practices, reducing the likelihood of OTA contamination (Van Egmond, 2004).

The report by Duarte and colleagues highlights how technological advancements in the brewing industry can significantly reduce Ochratoxin A (OTA) levels. This encompasses enhanced techniques for identifying and eliminating impurities, improved storage and handling measures to inhibit fungal development (a key OTA source), and general improvements in the manufacturing process, resulting in a purer and safer end product (Duarte, Pena, & Lino, 2010).

In their study, Mahdavi et al. discovered that every non‐alcoholic beer sample from Iran contained OTA (Mahdavi et al., 2007). Aragua et al. noted higher mean values and OTA contamination incidence in non‐alcoholic beer samples than in alcoholic ones. This difference could be linked to varying production processes; the absence of fermentation in non‐alcoholic beer production may contribute to elevated contamination levels if OTA is removed during fermentation (Araguás et al., 2005).

Accordingly, in Běláková et al.'s study, the analysis of OTA transmission during beer production involved samples of barley, malt, sweet wort, hopped wort, and beer. Barley varieties Bojos and Sebastian were treated chemically (fungicide) or untreated. No OTA was detected in barley and malt samples. Trace amounts were found in sweet wort from the variety Sebastian, possibly due to visible mold formation during refrigerated storage for 72 h, indicating potential contamination during lowered temperature storage. OTA was absent in the control wort sample and the beer from the variety Sebastian. Minimal OTA was detected in beer from the variety Bojos, suggesting that proper technological procedures can eliminate secondary fungal contamination; other samples had OTA levels below the limit of detection (Běláková et al., 2011).

As part of technological and procedural improvements, better control and screening of raw materials (like barley, hops, and water) used in beer production may also be possible. Preventing OTA contamination at the source is often more effective than addressing it later in production (Bertuzzi et al., 2011; Tangni & Larondelle, 2002). A more informed consumer base demanding higher‐quality, safer products can drive changes in industry practices. This consumer‐driven demand encourages manufacturers to adopt better practices to ensure lower product OTA levels (Aquilani et al., 2015; Villacreces et al., 2022).

### 3.2 Health risk assessment

**FIGURE 3:**
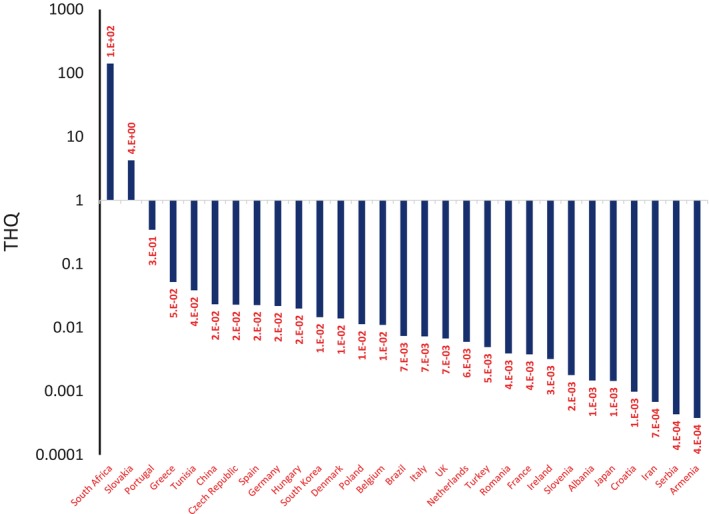
Target hazard quotient (THQ) in adult consumers due to OTA in beers.

Target hazard quotient (THQ) in adults of South Africa (THQ = 1.E+02) and Slovakia (THQ = 4.E+00) was higher than 1 value, but THQ in the other countries was lower than 1 value (Figure 3). The reasons for the variations in THQ values among different countries could be attributed to differences in exposure levels, dietary habits, environmental conditions, or the presence of the contaminant in various sources. Understanding the specific factors contributing to higher THQ values in South Africa and Slovakia would require a detailed analysis of the exposure pathways, population characteristics, and contaminant levels in the respective environments (Odhav & Naicker, 2002). Countries have diverse dietary patterns, which can influence the types and amounts of contaminants consumed. The consumption of certain locally produced foods, traditional dishes, or specific food processing methods can contribute to variations in exposure to contaminants, affecting THQ values. For example, in Slovakia, Branislav and his team observed that Slovakia leads in beer consumption, as evidenced by the increasing number of breweries. These breweries produce almost 3 million hectoliters of beer each year. Notably, around 60% of this production is consumed during the summer. Slovakia's beer is popular, with an average annual consumption of 73 liters per person. This figure places Slovaks among nations with a strong beer‐drinking culture, a trend that is on the rise. In terms of preferences, light beer is most popular among Slovaks (53.3%), followed by dark beer (23.1%) and Radler (15.3%) (Dudić et al., 2018).

The levels of contamination exposure can differ greatly among countries due to factors like industrial activities, agricultural practices, and pollution levels. These factors can lead to higher exposure in certain regions, affecting THQ values. For instance, Park et al. found that OTA in polished rice accounted for over 90% of total intake in their study, as this rice had the highest levels among the foods tested and is consumed by Koreans at a much higher rate than other local foods. Other significant sources of Ochratoxin A in Korea are barley and beer (Park et al., 2005). The primary contributors to Ochratoxin A consumption can vary internationally due to substantial differences in national and regional food consumption patterns. In Europe and North America, the main dietary sources of Ochratoxin A are cereal products (like flour‐based foods) and wine, with smaller amounts coming from grape juice, coffee, and dried vine fruits (Joint FAO/WHO Expert Committee on Food Additives Meeting and World Health Organization, 2001; Piron & Poelmans, 2016).

As indicated by Schabo et al., differences in the analytical methods used to measure contaminant levels can contribute to variations in THQ values. Variability in monitoring and testing practices between countries may affect the accuracy of exposure assessments (Schabo, Freire, et al., 2021). Considering the concurrent existence of diverse mycotoxins, such as simultaneous determination of OTA, aflatoxins, zearalenone, deoxynivalenol, fumonisins (fumonisin B1 and fumonisin B2 [FB1 and FB2]), T‐2 and HT‐2 toxins, and their potential toxicity even at low levels, it is crucial to utilize accurate and reliable methods to ensure the safety of the final product and compliance with regulations (Soleimany et al., 2012).

Xu et al. utilized the margin of exposure (MOE) method to evaluate OTA's nonneoplastic and neoplastic effects. In diverse exposure scenarios, MOE1 and MOE2 surpassed 200 and 10,000, respectively, reducing health risks. It is advisable to assess the well‐being of heavy drinkers and children, incorporating individual mycotoxin risk assessments. Urgent actions are warranted to oversee and regulate mycotoxins in beer. Despite the common practice of assessing risks for single substances, individuals face simultaneous exposure to multiple mycotoxins. This underscores the importance of comprehensively evaluating cumulative health risks posed by *Fusarium* mycotoxins, including DONs, nivalenol (NIV), ZEN, and FB1 (Xu et al., 2023). It is important to note that OTA is a known mycotoxin with potential health risks, including nephrotoxicity and carcinogenicity, making its presence in food and beverages, even at low levels, a significant public health concern. The finding underscores the need for improved agricultural storage, brewing practices, and stringent monitoring and regulation to ensure consumer safety (Běláková et al., 2011). The use of artificial intelligence (AI) in agriculture and the Internet of Things (IoT) in the storage process of cereals can be the basis for reducing mycotoxin contamination (Lutz & Coradi, 2022).

## 4 CONCLUSION

In the current study, the levels and prevalence of OTA in beer were meta‐analyzed, and the health risk in beer consumers was estimated. The highest prevalence of OTA in beer was observed in Armenia, the Netherlands, and the UK, and based on the levels of OTA in South Africa, Slovakia, and Portugal. Except for consumers in South Africa and Slovakia, non‐carcinogenic risk in other countries was acceptable due to OTA in beer. Therefore, it is recommended that effective beer quality control programs be carried out in South Africa and Slovakia. Hence, conducting more studies on the mycotoxins in beverages, especially traditional beverages in these countries, is recommended. Meta‐regression shows that the prevalence of OTA in beer decreased significantly over time, which shows the effectiveness of beer quality control plans globally. Finally, carrying out comprehensive quality control programs from raw materials to final products and improvement methods for determining the level of contamination can provide the basis for reducing the health risks due to OTA in beer.

## AUTHOR CONTRIBUTIONS

Search in databases was conducted by Yadolah Fakhri and Vahid Ranaei; Data collection by Yadolah Fakhri and Mansour Sarafraz; Data analysis by Yadolah Fakhri, and the manuscript and editing by Amin Mousavi Khaneghah, Yadolah Fakhri, Vahid Ranaei, Zahra Pilevar, Olga F. Belaia, Natalia V. Kolaeva and Mansour Sarafraz.

## CONFLICT OF INTEREST STATEMENT

The authors declare no conflict of interest relevant to this study.

## CONSENT TO PARTICIPATE

The authors declare their consent to participate in this study.

## CONSENT TO PUBLISH

The authors declare their consent to publish this study.

## Supporting information


Appendix S1.

Appendix S2.

## Data Availability

Not applicable.
