# Supplementary material for: Prevalence and concentration of Ochratoxin A in beer: A global systematic review, meta‐analysis, and health risk assessment
Source: Food Sci Nutr. 2024 Sep 10;12(11):8503–14. doi: 10.1002/fsn3.4456 (PMC11606901; doi:10.1002/fsn3.4456)
Supplement: Supplementary file 1 — Appendix S1. [file FSN3-12-8503-s001.docx]

**Appendix1.** Main characteristic included in our study

| **Study year** | **Country** | **Total sample size** | **Positive sample size** | **Mean of mycotoxins levels** | **SD** | **Analytical detection method**** | **LOD (ng/mL)** | **LOQ (ng/mL)** | **References** |
| --- | --- | --- | --- | --- | --- | --- | --- | --- | --- |
| 2000 | Italy | 61 | 30 | 0.035 | 0.031 | HPLC-FLD | 0.01 | NM | (Visconti et al., 2000) |
| 2001 | Morocco | 5 | 0 |  |  | HPLC-FLD | NM | 0.01 | (Filali et al., 2001) |
| 2001 | South Africa | 35 | 10 | 1170.750 | 584.625 |  | 1 | NM | (Filali et al., 2001) |
| 2002 | Belgium | 82 | 80 | 0.100 | 0.045 | HPLC-MS | 0.003 | 0.01 | (Tangni et al., 2002) |
| 2003 | Brazil | 30 | 8 | 0.055 | 0.013 | HPLC-FLD | 0.002 | 0.008 | (Prado et al., 2003) |
| 2004 | Turkey | 150 | 45 | 4.100 | 2.000 | ELISA | 0.08 | NM | (Gumus et al., 2004) |
| 2005 | Spain | 31 | 24 | 0.044 | 0.047 | HPLC-FLD | 0.012 | 0.02 | (Araguás et al., 2005) |
| 2005 | Italy | 18 | 8 | 0.080 | 0.030 | HPLC-MS | 0.01 | 0.03 | (Bacaloni et al., 2005) |
| 2005 | Taiwan | 18 | 0 |  |  | HPLC-FLD | 0.2 | NM | (Lin et al., 2005) |
| 2005 | Spain | 88 | 73 | 0.046 | 0.048 | HPLC-FLD | 0.005 | NM | (Medina et al., 2006) |
| 2005 | South Korea | 46 | 2 | 0.200 | 0.025 | HPLC-FLD | 0.2 | NM | (Park et al., 2005) |
| 2005 | Hungary | 25 | 25 | 0.127 | 0.055 | HPLC-FLD | 0.01 | NM | (Varga et al., 2013) |
| 2006 | Belgium | 80 | 71 | 0.103 | 0.162 | HPLC-FLD | 0.005 | 0.018 | (Anselme et al., 2006) |
| 2006 | Spain | 69 | 69 | 0.070 | 0.123 | HPLC-FLD | 0.001 | 0.003 | (Medina et al., 2006) |
| 2006 | Japan | 20 | 12 | 0.020 | 0.010 | HPLC-MS | 6.6 | 21 | (Sugita-Konishi et al., 2006) |
| 2006 | Brazil | 123 | 5 | 0.300 | 4.250 | HPLC-MS | 0.26 | NM | (Sugita-Konishi et al., 2006) |
| 2007 | South Africa | 48 | 13 | 0.075 | 0.003 | HPLC-FLD | 0.1 | 0.5 | (Maenetje et al., 2007) |
| 2007 | Iran | 70 | 70 | 0.260 | 0.130 | ELISA | 0.025 | NM | (Mahdavi et al.) |
| 2008 | Japan | 20 | 14 | 0.019 | 0.010 | HPLC-MS | NM | 0.1 | (Kumagai et al., 2008) |
| 2009 | Turkey | 35 | 5 | 0.020 | 0.014 | HPLC-FLD | 1 | 5 | (Kabak, 2009) |
| 2011 | Czech Republic | 115 | 28 | 0.120 | 0.060 | UHPLC-FLD | NM | NM | (Běláková et al., 2011) |
| 2011 | Albania | 4 | 3 | 0.015 | 0.014 | HPLC-FLD | 0.002 | 0.005 | (Běláková et al., 2011) |
| 2011 | Armenia | 2 | 2 | 0.033 | 0.005 | HPLC-FLD | 0.002 | 0.005 | (Běláková et al., 2011) |
| 2011 | Belgium | 7 | 6 | 0.016 | 0.016 | HPLC-FLD | 0.002 | 0.005 | (Běláková et al., 2011) |
| 2011 | Bosnia | 1 | 0 |  |  | HPLC-FLD | 0.002 | 0.005 | (Běláková et al., 2011) |
| 2011 | Croatia | 11 | 2 | 0.005 | 0.008 | HPLC-FLD | 0.002 | 0.005 | (Běláková et al., 2011) |
| 2011 | Czech Republic | 2 | 1 | 0.011 | 0.013 | HPLC-FLD | 0.002 | 0.005 | (Běláková et al., 2011) |
| 2011 | Denmark | 5 | 5 | 0.101 | 0.068 | HPLC-FLD | 0.002 | 0.005 | (Běláková et al., 2011) |
| 2011 | France | 5 | 4 | 0.009 | 0.006 | HPLC-FLD | 0.002 | 0.005 | (Běláková et al., 2011) |
| 2011 | Germany | 9 | 9 | 0.017 | 0.010 | HPLC-FLD | 0.002 | 0.005 | (Běláková et al., 2011) |
| 2011 | Ireland | 4 | 4 | 0.015 | 0.007 | HPLC-FLD | 0.002 | 0.005 | (Běláková et al., 2011) |
| 2011 | Italy | 17 | 11 | 0.007 | 0.006 | HPLC-FLD | 0.002 | 0.005 | (Běláková et al., 2011) |
| 2011 | Kosovo | 1 | 1 |  |  | HPLC-FLD | 0.002 | 0.005 | (Běláková et al., 2011) |
| 2011 | Macedonia | 1 | 1 |  |  | HPLC-FLD | 0.002 | 0.005 | (Běláková et al., 2011) |
| 2011 | Montenegro | 1 | 1 |  |  | HPLC-FLD | 0.002 | 0.005 | (Běláková et al., 2011) |
| 2011 | Poland | 4 | 3 | 0.046 | 0.047 | HPLC-FLD | 0.002 | 0.005 | (Běláková et al., 2011) |
| 2011 | Romania | 11 | 6 | 0.017 | 0.021 | HPLC-FLD | 0.002 | 0.005 | (Běláková et al., 2011) |
| 2011 | Russia | 1 | 1 |  |  | HPLC-FLD | 0.002 | 0.005 | (Běláková et al., 2011) |
| 2011 | Serbia | 8 | 2 | 0.003 | 0.003 | HPLC-FLD | 0.002 | 0.005 | (Běláková et al., 2011) |
| 2011 | Slovenia | 2 | 1 | 0.010 | 0.012 | HPLC-FLD | 0.002 | 0.005 | (Běláková et al., 2011) |
| 2011 | Spain | 2 | 2 | 0.031 | 0.009 | HPLC-FLD | 0.002 | 0.005 | (Běláková et al., 2011) |
| 2011 | Sweden | 1 | 1 |  |  | HPLC-FLD | 0.002 | 0.005 | (Běláková et al., 2011) |
| 2011 | Switzerland | 1 | 0 |  |  | HPLC-FLD | 0.002 | 0.005 | (Běláková et al., 2011) |
| 2011 | Netherlands | 2 | 2 | 0.042 | 0.009 | HPLC-FLD | 0.002 | 0.005 | (Běláková et al., 2011) |
| 2011 | Turkey | 2 | 2 | 0.014 | 0.003 | HPLC-FLD | 0.002 | 0.005 | (Běláková et al., 2011) |
| 2011 | UK | 2 | 2 | 0.043 | 0.038 | HPLC-FLD | 0.002 | 0.005 | (Běláková et al., 2011) |
| 2011 | China | 20 | 0 |  |  | HPLC-MS | 0.201 | NM | (Wu et al., 2011) |
| 2011 | Cameroon | 14 | 0 |  |  | HPLC-MS | ND | NM | (Wu et al., 2011) |
| 2013 | France | 51 | 9 | 0.092 | 0.044 | HPLC-FLD | 0.005 | NM | (Deetae et al., 2013) |
| 2013 | Tunisia | 35 | 17 | 0.120 | 0.082 | HPLC-FLD | 0.01 | 0.003 | (Lasram et al., 2013) |
| 2013 | Italy | 30 | 5 | 0.350 | 0.060 | HPLC-MS | 0.03 | 0.05 | (Prelle et al., 2013) |
| 2013 | Czech Republic | 24 | 22 | 0.135 | 0.063 | HPLC-FLD | NM | NM | (Skarkova et al., 2013) |
| 2013 | Malawi | 9 | 0 |  |  | HPLC-FLD | NM | NM | (Skarkova et al., 2013) |
| 2015 | Czech Republic | 132 | 107 | 0.024 | 0.023 | UHPLC-FLD | NM | 0.4 | (Běláková et al., 2011) |
| 2016 | Czech Republic | 49 | 44 | 0.060 | 0.298 | HPLC | 0.003 | 0.01 | (Lhotská et al., 2016) |
| 2016 | Czech Republic | 10 | 9 | 20.800 | 20.175 | HPLC-FLD | NM | 0.001 | (Lhotská et al., 2016) |
| 2016 | Poland | 12 | 7 | 6.700 | 12.025 | HPLC-FLD | NM | 0.001 | (Lhotská et al., 2016) |
| 2016 | Slovakia | 8 | 7 | 31.300 | 19.150 | HPLC-FLD | NM | 0.001 | (Lhotská et al., 2016) |
| 2016 | Tunisia | 34 | 1 | 2.000 | 1.040 | HPLC-FLD | 2 | 3.5 | (Lhotská et al., 2016) |
| 2016 | South Africa | 32 | 0 |  |  | HPLC-FLD | 5.2 | 10 | (Lhotská et al., 2016) |
| 2018 | Italy | 83 | 38 | 0.007 | 0.013 | HPLC-FLD | 0.001 | 0.003 | (Bertuzzi et al., 2011) |
| 2018 | Latvia | 100 | 0 |  |  | HPLC-FLD | 0.14 | 0.45 | (Bertuzzi et al., 2011) |
| 2019 | Poland | 69 | 64 | 0.057 | 0.065 | HPLC-FLD | 0.003 | 0.011 | (Grajewski et al., 2019) |
| 2020 | Greece | 31 | 11 | 0.707 | 0.368 | ELISA | 1 | 2–25 | (Batrinou et al., 2020) |
| 2020 | Portugal | 85 | 9 | 3.140 | 4.090 | HPLC-FLD | 0.14 | 0.43 | (Silva et al., 2020) |
| 2020 | Spain | 40 | 8 | 1.830 | 1.000 | HPLC-FLD | 0.06 | 0.2 | (Silva et al., 2020) |
| 2020 | Germany | 140 | 7 | 0.176 | 0.062 | HPLC-FLD | NM | 0.05 | (Silva et al., 2020) |
| 2021 | Spain | 10 | 8 | 1.830 | 1.180 | DLLME-MS/MS | NM | NM | (Carballo et al., 2021) |
| 2021 | Spain | 40 | 8 | 1.830 | 1.000 | DLLME-MS/MS | NM | NM | (Carballo et al., 2021) |
| 2023 | China | 158 | 0 | 0.354 | 0.184 | UHPLC-MS/MS | 0.5 | 1.5 | (Li et al., 2023) |

References

Anselme, M., Tangni, E., Pussemier, L., Motte, J.-C., Van Hove, F., Schneider, Y.-J., . . . Larondelle, Y. (2006). Comparison of ochratoxin A and deoxynivalenol in organically and conventionally produced beers sold on the Belgian market. *Food additives and contaminants, 23*(9), 910-918.

Araguás, C., González-Peñas, E., & De Cerain, A. L. (2005). Study on ochratoxin A in cereal-derived products from Spain. *Food Chemistry, 92*(3), 459-464.

Bacaloni, A., Cavaliere, C., Faberi, A., Pastorini, E., Samperi, R., & Laganà, A. (2005). Automated on-line solid-phase extraction− liquid chromatography− electrospray tandem mass spectrometry method for the determination of ochratoxin A in wine and beer. *Journal of agricultural and food chemistry, 53*(14), 5518-5525.

Batrinou, A., Houhoula, D., & Papageorgiou, E. (2020). Rapid detection of mycotoxins on foods and beverages with enzyme-linked immunosorbent assay. *Quality Assurance and Safety of Crops & Foods, 12*(1), 40-49.

Běláková, S., Benešová, K., Mikulíková, R., & Svoboda, Z. (2011). Determination of ochratoxin A in brewing materials and beer by ultra performance liquid chromatography with fluorescence detection. *Food Chemistry, 126*(1), 321-325.

Bertuzzi, T., Rastelli, S., Mulazzi, A., Donadini, G., & Pietri, A. (2011). Mycotoxin occurrence in beer produced in several European countries. *Food Control, 22*(12), 2059-2064.

Carballo, D., Fernández-Franzón, M., Ferrer, E., Pallarés, N., & Berrada, H. (2021). Dietary exposure to mycotoxins through alcoholic and non-alcoholic beverages in Valencia, Spain. *Toxins, 13*(7), 438.

Deetae, P., Perello, M. C., & De Revel, G. (2013). Occurrence of ochratoxin A and biogenic amines in Asian beers sold in French markets. *Journal of the Institute of Brewing, 119*(1-2), 57-63.

Filali, A., Ouammi, L., Betbeder, A., Baudrimont, I., Soulaymani, R., Benayada, A., & Creppy, E. (2001). Ochratoxin A in beverages from Morocco: a preliminary survey. *Food additives and contaminants, 18*(6), 565-568.

Grajewski, J., Kosicki, R., Twarużek, M., & Błajet-Kosicka, A. (2019). Occurrence and risk assessment of mycotoxins through polish beer consumption. *Toxins, 11*(5), 254.

Gumus, T., Arici, M., & Demirci, M. (2004). A survey of barley, malt and beer contamination with ochratoxin A in Turkey. *Journal of the Institute of Brewing, 110*(2), 146-149.

Kabak, B. (2009). Ochratoxin A in cereal-derived products in Turkey: occurrence and exposure assessment. *Food and Chemical Toxicology, 47*(2), 348-352.

Kumagai, S., Nakajima, M., Tabata, S., Ishikuro, E., Tanaka, T., Norizuki, H., . . . Kai, S. (2008). Aflatoxin and ochratoxin A contamination of retail foods and intake of these mycotoxins in Japan. *Food additives and contaminants, 25*(9), 1101-1106.

Lasram, S., Oueslati, S., Chebil, S., Mliki, A., & Ghorbel, A. (2013). Occurrence of ochratoxin A in domestic beers and wines from Tunisia by immunoaffinity clean-up and liquid chromatography. *Food Additives and Contaminants: Part B, 6*(1), 1-5.

Lhotská, I., Šatínský, D., Havlíková, L., & Solich, P. (2016). A fully automated and fast method using direct sample injection combined with fused-core column on-line SPE–HPLC for determination of ochratoxin A and citrinin in lager beers. *Analytical and bioanalytical chemistry, 408*, 3319-3329.

Li, X., Li, L., Zhou, Z., Li, T., An, J., Zhang, S., . . . Jia, Y. (2023). Soil potentially toxic element pollution at different urbanization intensities: Quantitative source apportionment and source-oriented health risk assessment. *Ecotoxicology and Environmental Safety, 251*, 114550.

Lin, L.-C., Chen, P.-C., Fu, Y.-M., & Shih, D.-C. (2005). Ochratoxin A contamination in coffees, cereals, red wines and beers in Taiwan. *Journal of Food and Drug Analysis, 13*(1), 12.

Maenetje, P. W., & Dutton, M. F. (2007). The incidence of fungi and mycotoxins in South African barley and barley products. *Journal of Environmental Science and Health Part B, 42*(2), 229-236.

Mahdavi, R., Khorrami, S. A. H., & Jabbari, M. V. VPF Research Journal of Biological Sciences 2 (5): 546-550, 2007.

Medina, Á., Valle-Algarra, F. M., Mateo, R., Gimeno-Adelantado, J. V., Mateo, F., & Jiménez, M. (2006). Survey of the mycobiota of Spanish malting barley and evaluation of the mycotoxin producing potential of species of Alternaria, Aspergillus and Fusarium. *International journal of food microbiology, 108*(2), 196-203.

Park, J. W., Chung, S.-H., & Kim, Y.-B. (2005). Ochratoxin A in Korean food commodities: occurrence and safety evaluation. *Journal of agricultural and food chemistry, 53*(11), 4637-4642.

Prado, G., Oliveira, M. S., Carvalho, E. P., Lima, L. C. O., Veloso, T., Souza, L. A. F., & Cardoso, A. C. F. (2003). Ochratoxin A determination in beer by immunoaffinity column clean-up and high-performance liquid chromatography. *Food Science and Technology, 23*, 58-61.

Prelle, A., Spadaro, D., Denca, A., Garibaldi, A., & Gullino, M. L. (2013). Comparison of clean-up methods for ochratoxin A on wine, beer, roasted coffee and chili commercialized in Italy. *Toxins, 5*(10), 1827-1844.

Silva, L. J., Teixeira, A. C., Pereira, A. M., Pena, A., & Lino, C. M. (2020). Ochratoxin A in beers marketed in Portugal: occurrence and human risk assessment. *Toxins, 12*(4), 249.

Skarkova, J., Ostry, V., Malir, F., & Roubal, T. (2013). Determination of ochratoxin A in food by high performance liquid chromatography. *Analytical Letters, 46*(10), 1495-1504.

Sugita-Konishi, Y., Nakajima, M., Tabata, S., Ishikuro, E., Tanaka, T., Norizuki, H., . . . Kai, S. (2006). Occurrence of aflatoxins, ochratoxin A, and fumonisins in retail foods in Japan. *Journal of food protection, 69*(6), 1365-1370.

Tangni, E., & Larondelle, Y. (2002). *Malts, moulds and mycotoxins.* Paper presented at the Bacteria, Yeasts and Moulds in Malting and Brewing: Proceedings of the Xth Symposium “Chair J de Clerck”, Leuven (Belgium).

Varga, E., Malachova, A., Schwartz, H., Krska, R., & Berthiller, F. (2013). Survey of deoxynivalenol and its conjugates deoxynivalenol-3-glucoside and 3-acetyl-deoxynivalenol in 374 beer samples. *Food Additives & Contaminants: Part A, 30*(1), 137-146.

Visconti, A., Pascale, M., & Centonze, G. (2000). Determination of ochratoxin A in domestic and imported beers in Italy by immunoaffinity clean-up and liquid chromatography. *Journal of chromatography A, 888*(1-2), 321-326.

Wu, J., Tan, Y., Wang, Y., & Xu, R. (2011). Occurrence of ochratoxin A in wine and beer samples from China. *Food additives and contaminants, 4*(1), 52-56.
