# Supplementary material for: Prevalence and concentration of Ochratoxin A in beer: A global systematic review, meta‐analysis, and health risk assessment
Source: Food Sci Nutr. 2024 Sep 10;12(11):8503–14. doi: 10.1002/fsn3.4456 (PMC11606901; doi:10.1002/fsn3.4456)
Supplement: Supplementary file 2 — Appendix S2. [file FSN3-12-8503-s002.docx]

**Appendix 2.** Ingestion rate of beer per countries (Adekoya et al., 2018; Helgilibrary, 2020; Matumba et al., 2011)

| **Country** | **IR (L/d)** |
| --- | --- |
| Albania | 0.117 |
| Armenia | 0.014 |
| Belgium | 0.182 |
| Brazil | 0.160 |
| China | 0.078 |
| Croatia | 0.234 |
| Czech Republic | 0.384 |
| Denmark | 0.164 |
| France | 0.090 |
| Germany | 0.271 |
| Greece | 0.088 |
| Hungary | 0.186 |
| Iran | 0.0031 |
| Ireland | 0.255 |
| Italy | 0.096 |
| Japan | 0.091 |
| Netherlands | 0.170 |
| Poland | 0.255 |
| Portugal | 0.131 |
| Romania | 0.275 |
| Serbia | 0.173 |
| Slovakia | 0.162 |
| Slovenia | 0.213 |
| South Africa | 0.144 |
| South Korea | 0.087 |
| Spain | 0.137 |
| Tunisia | 0.044 |
| Turkey | 0.031 |
| UK | 0.186 |

Reference

Adekoya, I., Obadina, A., Adaku, C. C., De Boevre, M., Okoth, S., De Saeger, S., & Njobeh, P. (2018). Mycobiota and co-occurrence of mycotoxins in South African maize-based opaque beer. *International journal of food microbiology, 270*, 22-30.

Helgilibrary. (2020). Indicators/beer-consumption-per-capita. <https://www.helgilibrary.com/indicators/beer-consumption-per-capita/canada/#:~:text=Beer%20consumption%20per%20capita%20reached,of%2054.1%20litres%20in%202021>.

Matumba, L., Monjerezi, M., Khonga, E. B., & Lakudzala, D. D. (2011). Aflatoxins in sorghum, sorghum malt and traditional opaque beer in southern Malawi. *Food control, 22*(2), 266-268.
